# Mild hydrostatic pressure triggers oxidative responses in *Escherichia coli*

**DOI:** 10.1371/journal.pone.0200660

**Published:** 2018-07-17

**Authors:** Aurelie Guyet, Martyn Dade-Robertson, Anil Wipat, John Casement, Wendy Smith, Helen Mitrani, Meng Zhang

**Affiliations:** 1 The Centre for Bacterial Cell Biology, Institute for Cell and Molecular Biosciences, Newcastle University, Newcastle upon Tyne, United Kingdom; 2 School of Computing, Newcastle University, Newcastle upon Tyne, United Kingdom; 3 School of Architecture Planning and Landscape, Newcastle University, Newcastle upon Tyne, United Kingdom; 4 Bioinformatics Support Unit, Faculty of Medical Sciences, Newcastle University, Newcastle upon Tyne, United Kingdom; 5 School of Engineering, Newcastle University, Newcastle upon Tyne, United Kingdom; 6 Department of Applied Sciences, Northumbria University, Newcastle upon Tyne, United Kingdom; Baylor College of Medicine, UNITED STATES

## Abstract

Hydrostatic pressure is an important physical stimulus which can cause various responses in bacterial cells. The survival and cellular processes of *Escherichia coli* under hydrostatic pressures between 10 MPa and 110 MPa have been studied. However, understanding bacterial responses to moderately elevated pressure of up to 10 MPa is useful for a range of different applications including for example in smart and responsive materials. In this study, the genetic responses of *E*. *coli* K-12 MG1655 to 1 MPa pressure was examined using transcriptomic analysis by RNA-Seq. The results show that 101 genes were differentially expressed under 1 MPa pressure in *E*. *coli* cells, with 85 of them up-regulated. The analysis suggested that some genes were over expressed to adapt the increase of oxygen levels in our system, and several functional categories are involved including oxidative stress responses, Fe-S cluster assembly and iron acquisition. Two differentially expressed genes *azuC* and *entC* were further investigated using RT-qPCR, and GFP reported strains of those two genes were created, AG1319 (*P*_*azuC*_ *azuC-msfgfp*) and AG1321 (*P*_*entC*_ *entC-msfgfp*). A linear response of *azuC* expression was observed between 0 MPa to 1 MPa by monitoring the fluorescence signal of strain AG1319 (P_*azuC*_ *azuC-msfgfp*). This study is the first report to demonstrate the genetic response of bacterial cells under 1 MPa hydrostatic pressure, and provides preliminary data for creating pressure sensing bacterial strains for a wide range of applications.

## Introduction

Hydrostatic pressure is an important physical stimulus particularly in deep-sea environments. Microorganisms, which are able to grow at moderately higher than atmospheric pressures (0.1–10 MPa), are considered to be piezotolerants. Others require higher pressure, such as 10–50 MPa and even over 50 MPa, for optimal growth, and are referred to as piezophiles and hyperpiezophiles, respectively [1]. Studies have been performed to investigate the response and survival of microorganisms under hydrostatic pressure for various reasons. Food technology scientists have developed ultra-high pressure (≥ 100 MPa) processes which are used to inactivate pathogens, without altering the properties of food, as an alternative to temperature pasteurisation [2], which can alter taste and consistency. High-pressure treatments have a much smaller impact on vitamins and flavour molecules than high temperature treatments, and this means that processed foods maintain freshness. These benefits mean that, this process is widely used in vegetable, meat and drink production. However, some studies have shown that certain pathogens have a high-pressure resistance. Malone, for example, reported genes of *Escherichia coli* O157:H7 that are involved in High-Pressure Resistance [3]. These findings have caused major concern among food processors and regulatory agencies. Most studies of hydrostatic pressure in biological systems have been conducted between 10 MPa (equivalent to sea depth of 1 km) to 100 MPa [4]. Recently, several studies on the effect of elevated pressure (10 MPa) on bioprocesses were conducted which have demonstrated that 10 MPa could improve biomass and/or product biosynthesis productivity [2]. However, there are no publications available on how microorganisms respond to moderate elevated pressure (0.1 MPa to 1 MPa) at a molecular level. With the development of synthetic biology techniques, bacterial response to pressure also has wider potential applications. Many bacteria-based sensors have been engineered to respond to different stimulus such as chemicals or light [5]. In mammalian cells there are known pressure-sensing promoters responsible, for example, for bone remodelling [6, 7]. However, there are currently no characterised low elevated (above normal atmospheric) pressure sensing genes within bacteria and no engineered pressure sensing strains for mild elevated pressures. If a bacterial pressure sensor were produced it might have broad applications as, for example, a control in industrial chemical synthesis or for detecting mechanical changes in soil substrates for soil improvement [8]. There is a demand, therefore, to understand the effect of mild elevated pressure on bacteria and to generate an overall view of genetic responses to a range of the hydrostatic pressures.

To study the effect of mildly elevated pressures in more detail we started with the model bacterium *E*. *coli*. A number of studies have investigated hydrostatic pressure effects on *E*. *coli* in the last 20 years, although *E*. *coli* is not a bacterial species that ordinarily grows at high pressure. *E*. *coli* cells are not believed to have evolved specific adaptation mechanisms to high pressure so many changes caused by elevated hydrostatic pressures appear to overlap with the responses to other environmental stresses, such as temperature, pH and high oxygen levels etc. [2]. Several studies on the response of *E*. *coli* to a wide range of pressure below 100 MPa revealed that both heat shock proteins and cold shock proteins are up-regulated to adapt to the elevated pressure [9, 10]. Those studies also reported that elevated pressure changes the expression of the DNA-binding protein H-NS. It has been proven that H-NS protein is essential for cell growth under high-pressure conditions and likely to be a transcriptional regulator for genes related to the adaption of *E*. *coli* to high pressure [3]. In addition, studies show that high pressure and ultra-high pressure can induce oxidative stress and trigger an “SOS” response in *E*. *coli* [3, 11, 12]. Studies also suggested that high pressure could cause indirect DNA damage due to the activation of endonuclease, which, in turn, gives the signal to induce an SOS response in *E*. *coli* cells [11, 13].

This paper describes experimental work to investigate the genetic response of *E*. *coli* to mild elevated pressure (1 MPa) using transcriptomic analysis by RNA-Seq. The functions of genes involved in adaption of *E*. *coli* cells to 1 MPa were analysed to elucidate the potential physiological events under such pressure. The changes in expression level under pressure of two selected genes were confirmed using RT-qPCR and one gene was further characterised under a range of pressures between 0 MPa and 1 MPa.

## Materials and methods

### Strains and culture conditions

*E*. *coli* K-12 MG1655 was used for transcriptomics analysis of gene expression responding to mild elevated pressure (1 MPa), construction of GFP reporter strains to test pressure sensitivities of selected genes and as the negative control for pressure sensitivity experiments. *E*. *coli* K-12 MG1655 derived strain, HS524 (*mreB-msfgfp*) [14] was used in pressure sensitivity experiments as the positive control for GFP fluorescence signal. All bacterial strains and plasmids used in this study are listed in S1 Table.

*E*. *coli* K-12 MG1655, its derivatives and HS524 were grown shaking at 160 rpm at 37°C in LB-Miller broth (pH 7.4) or in M9 medium to minimise the GFP fluorescence background signal. M9 medium contained, 6 g/L of Na_2_HPO_4_ anhydrous, 3 g/L of KH_2_PO_4_, 0.5 g/L of NaCl, 1 g/L of NH_4_Cl, 0.4% (wt/vol) of glucose, 2 mM of MgSO_4_, 0.1% (wt/vol) of casamino acids and 0.1 mM of CaCl_2_. Strains were streaked on LB agar plates and grown in presence of ampicillin (100 μg/mL), chloramphenicol (12.5 μg/mL) or kanamycin (50 μg/mL), when needed.

### Transcriptomic response to low pressure exposure

A comparative transcriptomics experiment was conducted to observe the changes in gene expression of *E*. *coli* exposed to mild elevated pressure (1 MPa) in a bespoke pressure vessel (S1 Fig) which consisted of a stainless-steel chamber of an internal diameter of 6 cm, a lid with an input valve and a digital thermometer. The pressure vessel was connected to a cylinder of synthetic air consisting of 20%/80% oxygen/nitrogen pressurised to 20 MPa. The input valve of the compressed air cylinder was connected to a regulator with a maximum output pressure of 1 MPa.

Initially, 40 ml of *E*. *coli* culture was grown to an OD_600 nm_ of 0.7, and 1 ml aliquots placed into the pressure vessel vertically. The vessel was sealed and pressurized at 1 MPa for 15 min. The temperature within the chamber was recorded during periods of pressure treatment. The high-pressure samples (HP) refer to the tubes with modified caps with 1 mm holes drilled through the top, and the low-pressure samples (LP) refer to the tubes sealed with normal caps to protect them from pressure change. The samples were immediately removed and spun down for 1 min at 14,000 *x g* at room temperature (RT). The pellets were stored at -80°C in preparation for RNA extraction.

Total RNAs from two biological replicates, each with two technical replicates were extracted using the Total RNA Purification Kit (Norgen Biotek, UK) according to the manufacturer’s protocol with the addition of 1 mg/ml lysozyme for cell lysis. The lysates were applied to gDNA removal columns and rRNA was removed from total RNA using a Ribo-Zero rRNA removal kit (Illumina, UK) following the manufacture’s instruction for Gram-positive bacteria. To check the quality and quantity of the RNA, samples were analyzed using a Bioanalyser 2100 (Agilent Genomics, UK). The sequencing libraries were prepared from the enriched mRNAs using the NEBNext Ultra RNA library prep kit (NEB, UK), and independently indexed using NEBNext multi-plex oligos for Illumina (NEB, UK). The libraries were pooled at equimolar ratios before they were sequenced using MiSeq Reagent Kit v3 600 cycles (Illumina,UK).

The quality of sequencing data was assessed using FastQC, (V11.2) before the reads were trimmed using Trimmomatic (V0.33). The trimmed reads were aligned against *E*. *coli* MG1655 (GenBank: U00096.3) using bowtie2 (Version 2.2.4). The counts of reads aligning to genomic features were obtained using the 'featureCounts' function from the R package 'Rsubread'. The overall quality was then analysed through a principal component analysis (PCA). Gene annotation was obtained from the EcoCyc *E*. *coli* Database (https://ecocyc.org/). The R package 'DESeq2' (Version 1.12.4) was used to calculate differential gene expression between the LP group and the HP group. The genes with a Benjamini and Hochberg P-value of less than 0.05, log_2_ > 1.584 and where the difference in expression ≥ 3 fold were considered as differentially expressed.

In order to try to understand the main biological functions of the differentially expressed genes identified from RNA-Seq analysis, the differentially expressed genes were submitted to the Clusters of Orthologous Groups of proteins (COGs) database (https://www.ncbi.nlm.nih.gov/COG/) and Gene Ontology (GO) analysis (http://geneontology.org/). All RNA-Seq data were deposited to Gene Expression Omnibus Database under the accession number GSE114917.

### Confirmation of selected pressure sensitive targets using RT-qPCR

The functions of the differentially expressed genes were analysed, and two genes with fold changes (Fc) at different levels were selected for further study (S2 Table). To further investigate the RNA-Seq results, the expression of these two differentially expressed genes under 1 MPa pressure treatment were analysed using reverse transcription quantitative PCR (RT-qPCR). The housekeeping gene *rrsA* was chosen as a reference gene for the RT-qPCR experiment due to the stability of its expression under control and pressured conditions (data not shown). Primers binding to *rrsA*, *azuC* and *entC* (S2 Table) were designed using Primer3plus (http://www.bioinformatics.nl/cgi-bin/primer3plus/primer3plus.cgi). The pressure treatment and the total RNA extraction were performed as described in transcriptomic experiment with the addition of the no-pressure samples (NoP) which refer to samples placed outside the pressure vessel vertically. Then 0.5 μg of total RNA from each sample were converted into cDNA using the High-Capacity cDNA Reverse Transcription Kit with RNA inhibitor (Applied Biosystems, UK). cDNA were diluted at 1:60 and 6 μl were used in a 20 μl final PCR reaction using GoTaq qPCR Master Mix (Promega, UK). The reactions were performed in a Rotor-Gene Q real-time PCR cycler (Qiagen, UK), and the PCR cycler was set up with 95°C for 10 min, followed by 40 cycles of 95°C for 5 s and 60°C for 30 s. For the melting curves, a ramp from 50°C to 99°C was set up to rise 1°C each step, holding for 5 s at each step. Melt-curves analysis confirmed the efficiency and specificity of the primer pairs chosen. The threshold cycle (CT) values were obtained from the amplification curves, and the gene expression Fc was calculated using the 2^−ΔΔCT^ method [15]. The housekeeping gene *rrsA* was used as reference gene to normalize the qPCR data to cDNA input levels. RT-qPCR was performed with one biological sample for each pressure condition and set up with three technical replicates. *n*-fold change in transcript levels were normalized with respect to *rrsA* and to NoP samples.

### Creating pressure sensitive GFP reporter strains

To monitor the pressure sensitivity in the promoters of the selected differentially expressed genes (*azuC* and *entC*), the GFP reporter system was cloned into the targeted loci of the *E*. *coli* MG1655 chromosome to create a translational fusion. Firstly, we engineered two plasmids pAGcc3 and pAGcc4 (S1 Table and S2 Fig) carrying the features of interest flanked by the chromosomal DNA regions of the gene targeted. The assembled features were then amplified in a single linear DNA product which recombined to *E*. *coli* chromosome using the lambda red recombinase system to achieve targeted mutagenesis [16].

Plasmids pAGcc3 and pAGcc4 (S2 Fig) were obtained by Gibson assembly cloning and carry common features obtained by PCRs which are described hereafter, and all the primers are presented in S3 Table. Plasmid pBAD33 [17] was used as construct backbone (pBAD). The sequence was amplified using primers AG649-650. The 732 bp monomeric superfolder *gfp* gene was amplified from plasmid pHJS105 (*msfgfp*)[18]. A chloramphenicol resistance cassette (*cat*) containing 918 bp was amplified from pBAD33, whereas the 121 bp long terminator of transcription (T0) was amplified from pSEVA235. The primers used in the PCR reactions for *msfgfp*, *cat* and T0 were AG653-654, AG655-656 and AG 657–658, respectively. Chromosomal DNA of *E*. *coli* MG1655 was used as the template to amplify what were denoted as the upstream and downstream parts, which corresponded to the flanking DNA regions of locus of interest.

To engineer plasmid pAGcc3 (S2A Fig), a 586 bp upstream part was obtained by PCR, using primers AG651-652 to amplify *azuC* promoter and *azuC*. The 609 bp downstream part corresponding to the DNA located downstream *azuC* was amplified with primers AG659-660.

To create plasmid pAGcc4 (S2B Fig), the upstream part composed of the last 551 bp of *entC* coding DNA sequence was amplified from MG1655 chromosomal DNA using AG661-662 primers. As *entCEABH* is organized in one operon, pAGcc4 was designed to have the transcription of *entEABH* under the control of a second copy of *entC* promoter. The downstream part of pAGcc4 was composed of two chromosomal DNA regions. One was the promoter of *entC*, obtained using primers AG663-664 and was assembled downstream of the T0 part. The other chromosomal DNA was amplified with primers AG665-666 and carried 571 bp corresponding to a 5’ part of *entE* CDS which would allow recombination to the chromosome during the mutagenesis.

The above DNA parts were amplified by PCR using Q5 DNA polymerase (NEB, UK), and the residual template plasmids was removed by a *Dpn*I restriction digest. Gibson assembly reactions were performed using NEB Gibson Assembly® Cloning Kit (NEB, UK) according to the manufacturer’s instructions. The reaction mixture then was transformed into competent cells o*f E*. *coli* NEB 5α, and selected on LB agar plate containing 12.5 μg/ml chloramphenicol. The colonies were analysed by PCR reactions using selected primers in S3 Table, each positive clone carries all the plasmid parts. The plasmids were extracted and then confirmed by sequencing (GATC Biotech).

The translational fusion strains AG1319 (P_*azuC*_ *azuC*-*msfgfp*) and AG1321 (P_*entC*_ *entC*-*msfgfp*) were generated using the lambda red recombinase system [16]. The linear DNAs bearing the designed plasmid parts (*cat*, *msfgfp*, T0, upstream and downstream) were amplified from pAGcc3 and pAGcc4 using primers AG670-671 and then purified using the QIAquick PCR purification kit (Qiagen, UK) and transformed into *E*. *coli* MG1655 pKD46 electro-competent cells. The positive transformants were selected on LB-agar containing 12.5 μg/ml chloramphenicol at 30°C and afterwards were confirmed to be sensitive to ampicillin. Using PCR screening, positive mutants were identified as amplicon of 2107 bp and 2427 bp generated using primers AG677-678 (*azuC*) and AG675-676 (*entC*), respectively. The correct loci integrations were confirmed by sequencing (GATC Biotech) the PCR products. All the primers are presented in S3 Table.

### Characterisation of GFP responses of the engineered strains exposed to pressure

To measure the GFP responses to pressure, overnight cultures of strains MG1655, AG1319 and HS524 were diluted to an OD_600 nm_ of 0.04 and grown in M9 medium at 37°C for 3 hours. For each strain, 1 mL aliquots were treated under a range of pressures at 37°C for period of 3 hours. At end of treatment, three aliquots of 200 μl from each sample were transferred into a 96-well plate (Greiner BIO-ONE CELLSTAR, 655088), then the TECAN Spark^TM^ 10M multimode Microplate reader was used to monitor the optical cell density at OD_600 nm_ and the GFP signal with excitation 485 nm /emission filter 510 nm. *E*. *coli* strain HS524 was used as a positive control, as *mreB-msfgfp* expression was not sensitive to pressure in our tested conditions. The quantification of the signal, that is the relative fluorescence units per OD_600nm_ (RFU.OD_600nm_^-1^) was calculated using Eq [1]. This equation takes into account the noise from the medium as blank and the natural fluorescence of the wild type (wt) MG1655. The signal was normalised to the cells density for each strain.

$$RFU.{OD600nm}^{-1}=\frac{Avg.\;of\;(GFPstrain-GFP\;blank)}{Avg.\;of\;(OD600strain-OD600blank)}-\frac{Avg.of\;(GFPwt-GFP\;blank)}{Avg.\;of\;(OD\;600wt-OD600blank)}$$ Eq [1]

Six distinct assays were performed when strains were exposed to one of following pressure conditions: 0, 0.2, 0.4, 0.6, 0.8 and 1 MPa. Each assay contained three biological samples, with technical triplicates for each biological sample.

## Results

### Overview of transcriptomic response to low-pressure exposure

In order to study the *E*. *coli* genes response to mild elevated pressure, the *E*. *coli* cells were treated with 1 MPa pressure and the transcriptome of treated cells (HP) was compared with that from the control cells (LP) placed in the pressure vessel. Four samples from two biological cultures, each with two technical replicates were prepared for each condition. During the periods of pressure treatment, the temperatures in the pressure vessel were recorded (S3 Fig). Within 40 s after the pressure was applied, there was a 3.5°C increase from 26.7°C to 30.2°C, and then a gradual drop to 26.9°C for the duration of the experiment. As the vessel was depressurised, the temperature dropped briefly to 24.4°C degrees before the samples were removed for RNA extraction.

The principal component analysis (PCA) shows that up to 87% of the variance represented by PC1 was attributable to the pressure treatment and 4% of the variance represented by PC2 was attributable to the individual samples (S4 Fig). However, it is noticeable that one of the LP sample was much closer to counts for the HP samples. We suspected that there was a leak in this sample during the pressure treatment, so it was removed in the differential expression analysis.

**Fig 1:**
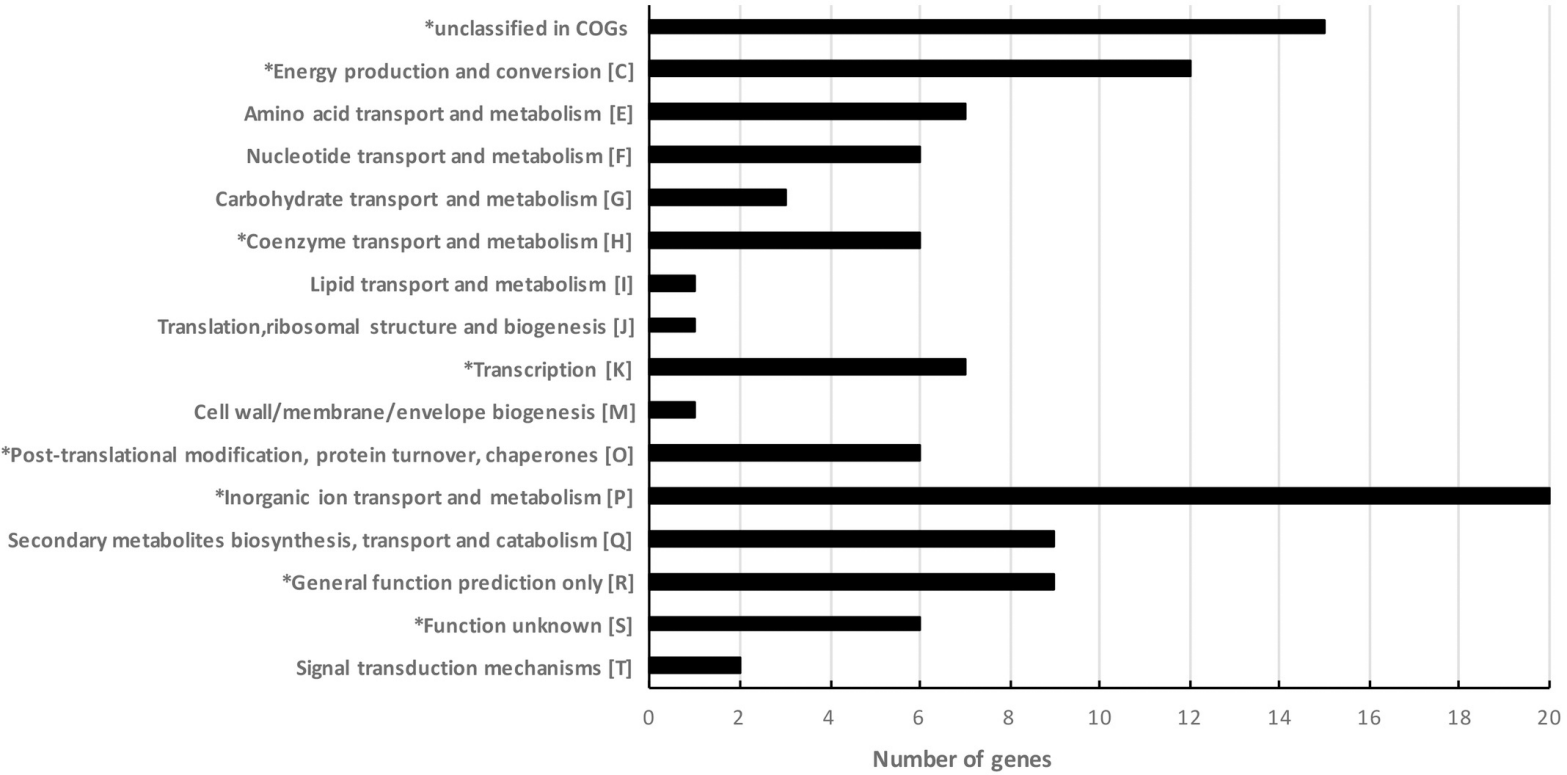
Analysis of the differentially expressed genes from RNA-Seq results using the COGs classification. *Contain genes down-regulated under 1MPa pressure. A total of 101 *E*. *coli* MG1655 genes identified as differentially expressed upon exposure to 1 MPa by RNA-Seq were represented here according to information available on the clusters of orthologous groups (COGs). There are 7 genes that belong to more than one COG groups and they were represented in each corresponding groups.

Overall, there were 101 genes that displayed a significant change in expression with a cut-off of Fc ≥ 3. Among these, 85 genes were up-regulated when the cells were exposed to 1 MPa up to 21-fold difference (S4 Table) and 16 genes down-regulated up to 9-fold (S5 Table). The functions of these genes were analysed using COG and GO classes. 101 genes were classified into 15 COG categories (Fig 1). As shown in Fig 1, 86 genes belong to at least one functional COG and among these 6 genes belong to the uncharacterized conserved protein group (S). In addition, 15 genes were not found in the COG classification, while as there are 7 genes belonging to more than one COG category (S4 & S5 Tables). Notably, a high number of genes (20) were identified up-regulated in the RNA-Seq as belonging to the inorganic ion transport and metabolism COG class (P) and 17 of these genes have an iron-related GO biological process. Indeed, there are 40 up-regulated genes under 1MPa involved in iron-related biological process (Table 1).

**Table 1:** Up-regulated genes involved in iron-related biological process.

Gene	Gene description	Fc	Operon	Roles
*fdx*	reduced ferredoxin	11.64	*hscBA-fdx-iscX*	Fe-S cluster biosynthesis
*hscA*	chaperone for [Fe-S] cluster biosynthesis	12.31	*hscBA-fdx-iscX*	Fe-S cluster biosynthesis
*hscB*	co-chaperone for [Fe-S] cluster biosynthesis	15.49	*hscBA-fdx-iscX*	Fe-S cluster biosynthesis
*iscX*	regulator of iron-sulfur cluster assembly	9.63	*hscBA-fdx-iscX*	Regulator
*iscA*	iron-sulfur cluster assembly protein	10.93	*iscRSUA*	Fe-S cluster biosynthesis
*iscS*	cysteine desulfurase	12.90	*iscRSUA*	Fe-S cluster biosynthesis
*iscU*	scaffold protein for iron-sulfur cluster assembly	10.62	*iscRSUA*	Fe-S cluster biosynthesis
*iscR*	IscR DNA-binding transcriptional dual regulator	12.64	*iscRSUA*	Regulator
cirA*	Outer membrane receptor for ferrienterochelin and colicins	21.3	*cirA*	Iron acquisition
efeB*	heme-containing peroxidase/deferrochelatase	6.40	*efeOB*	Iron acquisition
efeO*	periplasmic protein, component of cryptic ferrous ion transporter	8.44	*efeOB*	Iron acquisition
*entA*	2,3-dihydro-2,3-dihydroxybenzoate dehydrogenase	10.28	*entCEBAH*	Iron acquisition
*entB*	Enterobactin synthase component B	13.58	*entCEBAH*	Iron acquisition
*entC*	isochorismate synthase 1	12.71	*entCEBAH*	Iron acquisition
*entD*	Enterobactin synthase component D	3.76	*fepA-entD*	Iron acquisition
*entE*	2,3-dihydroxybenzoate-AMP ligase	14.77	*entCEBAH*	Iron acquisition
*entF*	holo [EntF peptidyl-carrier protein], apo-serine activating enzyme	13.72	*fes-ybdZ-entF-fepE*	Iron acquisition
*entH*	proofreading thioesterase in enterobactin biosynthesis	9.49	*entCEBAH*	Iron acquisition
entS*	Enterobactin exporter	8.19	*entS*	Iron acquisition
*fecA*	ferric citrate outer membrane porin FecA	13.16	*fecABCDE*	Iron acquisition
fecB*	ferric citrate ABC transporter—periplasmic binding protein	12.75	*fecABCDE*	Iron acquisition
fecC*	ferric citrate ABC transporter—membrane subunit	9.33	*fecABCDE*	Iron acquisition
fecD*	ferric citrate ABC transporter—membrane subunit	7.00	*fecABCDE*	Iron acquisition
fecE*	ferric citrate ABC transporter–ATP binding subunit	6.35	*fecABCDE*	Iron acquisition
*fecI*	RNA polymerase sigma 19 factor	4.24	*fecIR*	sigma factor
fecR*	regulator for fec operon	4.22	*fecIR*	Regulator
fepA*	ferric enterobactin / colicin B / colicin D outer membrane porin FepA	13.14	*fepA-entD*	Iron acquisition
fepB*	ferric enterobactin ABC transporter—periplasmic binding protein	5.95	*fepB*	Iron acquisition
fepC*	ferric enterobactin ABC transporter—ATP binding subunit	5.61	*fepDGC*	Iron acquisition
fepD*	ferric enterobactin ABC transporter—membrane subunit	3.80	*fepDGC*	Iron acquisition
fepG*	ferric enterobactin ABC transporter—membrane subunit	5.32	*fepDGC*	Iron acquisition
fes*	enterochelin esterase	5.84	*fes-ybdZ-entF-fepE*	Iron acquisition
fhuE*	ferric coprogen outer membrane porin FhuE	8.31	*fhuE*	Iron acquisition
*fhuF*	hydroxamate siderophore iron reductase	7.82	*fhuF*	Iron acquisition
fiu*	putative outer membrane receptor for iron transport	19.09	*fiu*	Iron acquisition
*ybdZ*	MbtH-like protein that enhances the catalytic function of EntF	3.54	*fes-ybdZ-entF-fepE*	Iron acquisition
*nrdH*	glutaredoxin-like protein	5.20	*nrdHIEF*	DNA replication under iron starvation
*nrdI*	flavodoxin involved in dimanganese-tyrosyl radical cofactor maintenance for ribonucleotide reductase	7.03	*nrdHIEF*	DNA replication under iron starvation
*nrdE*	ribonucleoside-diphosphate reductase 2, &alpha; subunit	12.93	*nrdHIEF*	DNA replication under iron starvation
*nrdF*	ribonucleoside-diphosphate reductase 2, &beta; subunit	9.26	*nrdHIEF*	DNA replication under iron starvation

*Genes belong to inorganic ion transport and metabolism COG class (P) and have an iron-related GO biological process.

### Validation of transcriptome sequencing results

After the functions of the differentially expressed genes were analysed, two genes, *azuC* and *entC*, were selected for validation and further pressure study. *azuC*, encodes a small membrane protein with no COG classification found. This gene was up-regulated more than 3-fold in the pressure treated cells. Literature has shown that this protein was also over expressed in presence of glucose, acidic pH, heat shock, oxidative stress, and thiol stress [19]. Another gene *entC* is involved in enterobactin synthesis and shown to have a ratio change at 12.7-fold in pressure treated cells (S4 Table).

**Fig 2:**
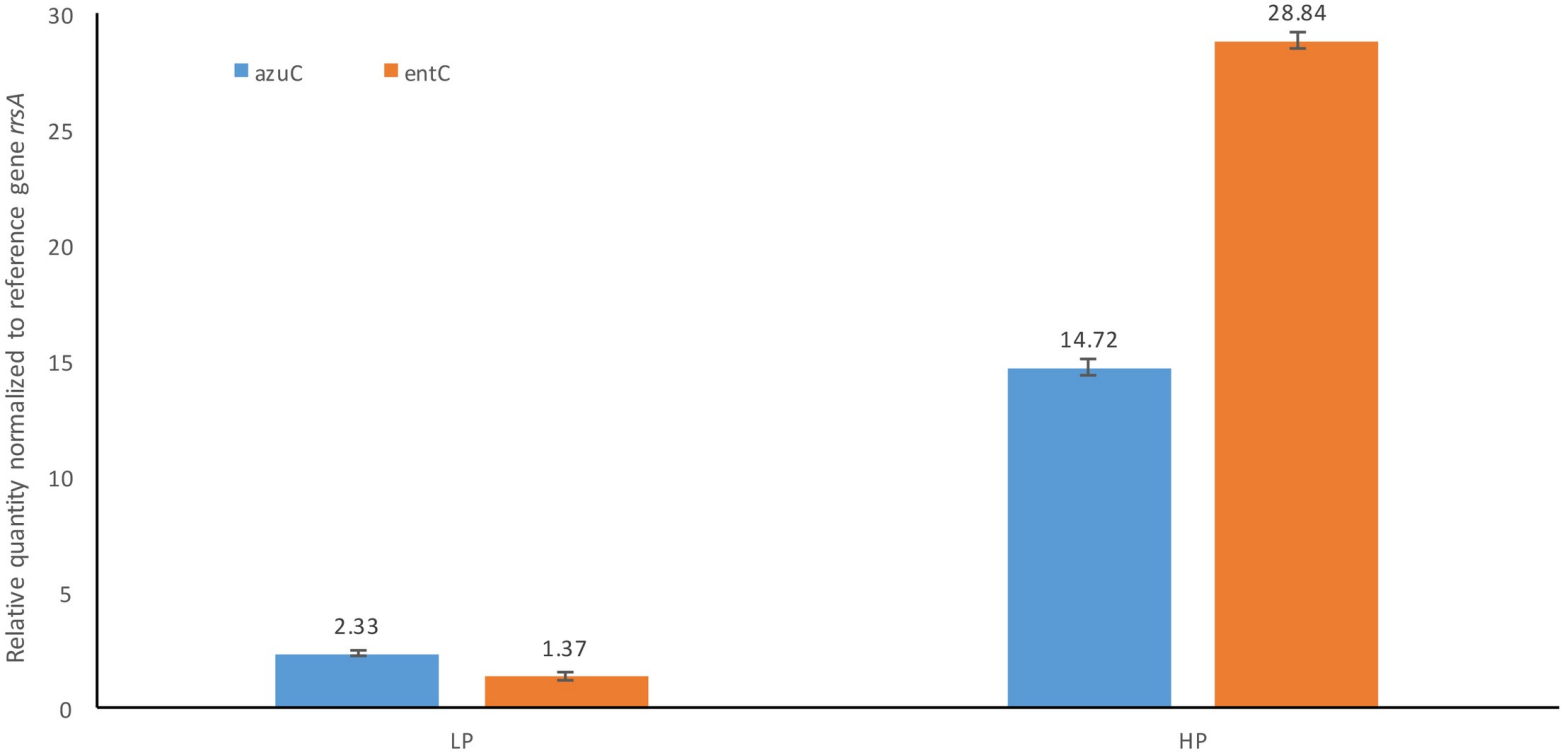
*azuC* and *entC* gene expression changes under 1 MPa analysed using RT-qPCR. HP refer to samples that were treated with 1 MPa, and LP refer to control samples inside the pressure vessel. *n*-fold change normalized to housekeeping gene *rrsA* and to NoP condition. Error bars, SD (n  =  3) from one biological culture with 3 technical replicates.

To further investigate the transcriptome sequencing results, RT-qPCR was used to quantify changes in the transcript levels of two selected genes, *azuC* and *entC*. The relative quantities of *azuC* and *entC* transcripts under 1 MPa (HP), which were normalized to the reference gene *rrsA* and to no pressure condition (NoP) using 2^−ΔΔCT^ method, were 14.72 and 28.84, respectively (Fig 2). These lead to 6.32-fold up-regulation on *azuC* expression and 21.05-fold up-regulation on *entC* expression when comparing with LP condition. Although discrepancies were observed, they were expected between RNA-Seq and RT-qPCR experiments as they rely on different data normalization methods. These results significantly validate the up-regulation of both genes observed in RNA-Seq experiment, therefore, they continued to be used in the pressure sensitive study.

### Characterisation of GFP responses of the engineered strains exposed to pressure

To monitor the pressure sensitivity in native promoters of *azuC* and *entC*, two translational fusion strains were created AG1319 (P_*azuC*_ *azuC-msfgfp*) and AG1321 (P_*entC*_ *entC-msfgfp*). GFP responses of AG1319 and AG1321 exposed to pressure of 1 MPa were tested along with that of MG1655 and HS524 strains where were used as negative and positive GFP control respectively. Significant difference in the pressure treated cells and control cells of AG1319 strain was mainly observed at 3 hours. However, the GFP signal changes in strain AG1321 was less significant in our tested condition (data not shown) and for this reason it was not further characterised.

To characterise the response of strain AG1319 to a range of pressures, cells were grown in M9 medium and were exposed to pressures of 0 MPa, 0.2 MPa, 0.4 MPa, 0.6 MPa, 0.8 MPa and 1 MPa. The RFU per OD_600nm_ at time 3 h treatment was used to calculate the ratio difference between pressure treatment and control conditions (Table 2). The ratio observed for HS524 strain remained around 1 at all pressure conditions. The coefficient of regression analysis is 0.0019 (p = 0.933). In contrast, strain AG1319 displayed the highest sensitivity at 1 MPa (ratio at 1.39) and remained pressure sensitive until 0.6 MPa (ratio at 1.16). The coefficient of regression analysis is 0.949 (p = 9.91E-4) and this demonstrates that AG1319 displayed mild pressure dose sensitivity.

**Table 2:** Strains response to different pressure after 3 h exposure to stress.

Pressure (MPa)	HS524 strain (*mreB-msfgfp*)			AG1319 strain (P_*azuC*_ *azuC-msfgfp)*		
	Under pressure^a^	No pressure^a^	Ratio^b^	Under pressure^a^	No pressure^a^	Ratio^b^
1	61820±1614	64899±5492	0.95	102853±3016	74030±1431	1.39
0.8	70135±2747	76886±4844	0.91	98872±2040	76948±2065	1.28
0.6	62292±5476	61260±4984	1.01	93261±1480	80157±1881	1.16
0.4	66540±9790	65870±10949	1.01	80713±3010	75110±5158	1.07
0.2	54629±9893	55812±9451	0.98	63962±6533	60707±3634	1.05
0	67521±4047	74915±9865	0.90	80655±2804	80918±3267	0.99

^a^Unit is RFU.OD_600nm_^-1^ and the values show the mean and standard deviation from three biological samples which were calculated using the formula shown in Eq [1].

^b^The ratios were calculated by the mean of RFU.OD_600nm_^-1^ of high pressure value/ the mean of RFU.OD_600nm_^-1^ of No pressure value.

## Discussion

The growth, survivability and adaptation of *E*. *coli* to pressure has been studied in terms of moderate and high hydrostatic pressure. It has been shown that elevated pressures progressively impair macromolecular synthesis starting with translation and then replication and transcription. It has been reported that DNA replication begins to become impaired at 50 MPa and ceases between 50–80 MPa. Transcription is suppressed at 20 MPa and inhibited at 80 MPa. Transcription can, however resume after decompression up to 180 MPa [20]. Similarly, growth has shown to be progressively inhibited but not stopped up to 50 MPa [21]. Cells also become filamentous under growth pressures, in part, due to the lack of the FtsZ protein [22] whose gene is down regulated at high pressure (40 MPa). While it seems that high pressure has a predominantly inhibitory effect on gene expression there are also indications of some genes which show increased expression in response to pressure changes [2, 3, 9, 10, 23]. However, all those studies were conducted under pressures of over 10 MPa, and pressures of between 0.1 MPa to 1 MPa have not yet been tested. The current study is the first work to study genetic response of *E*. *coli* to pressure at 1 MPa. Follonier [2] suggested that while pressures up to 1 MPa are too low to cause effects on molecular systems directly they may have indirect consequences such as inducing variations in dissolved gas concentrations. We found there were 101 genes differentially expressed when the cells were exposed to 1 MPa and among these, 85 genes were upregulated. The expression of many are related to the increase of oxygen levels in the system, but it is noteworthy that the majority of the up-regulated genes are involved in iron metabolism.

### Stress response related and oxygen level related genes

It is well documented that *E*. *coli* overexpress stress related genes under high-pressure conditions. The most discussed pressure related up-regulated proteins are heat shock proteins and cold shock proteins (Hsp and Csp, respectively). Csp was observed to be up-regulated from 10 to 100 MPa in several studies and heat shock proteins were observed to be up-relegated when the pressure was above 50 MPa [3, 9, 10]. It was proposed that high pressure may cause proteins to unfold within the cells and heat shock proteins are acting as protein chaperons to assist the protein refolding to maintain the correct activity. Meanwhile cold shock proteins help in maintaining energy metabolism [10], membrane fluidity [24] and facilitate accurate translation [9]. Although, we did not observe the changes in expression of well-known Hsps and Csps, interestingly, *azuC* was found to be up-regulated in our experiment. It was found down regulated under low oxygen levels, and over expressed under other stress conditions such as low pH, heat shock and oxidative stress [19]. The study suggested that along with other stress induced small membrane proteins, AzuC could play an important role in affecting membrane permeability and stabilizing the membrane by interacting with the inner membrane and modulating the function of other transmembrane proteins. It is tempting to speculate that AzuC was upregulated in response to mild elevated pressure to maintain membrane stability just like Csps in higher pressure treatments. We also found *hscA* and *hscB* were upregulated 12.31 and 15.49 fold respectively in our RNA-Seq experiments. These two genes encode for an Hsp70 type molecular and co-chaperone molecules. But Hesterkamp and Bukan [25] have proved that HscA was unable to replace DnaK, a major Hsp70 chaperon in *E*. *coli* to assist the folding and refolding of a range of proteins. Therefore, we believe that the up-regulation of these two genes are more related to the assembly of Fe-S clusters which will be discussed below.

Other stress related mechanisms which can be triggered by high pressure are the ROS and SOS responses. When the cells were treated with high pressure, an increase in oxygen solubility can generate oxidative stress and result in the accumulation of reactive oxygen species (ROS) causing damage to DNA [26]. Our RNA-Seq data has shown that the expression of SoxS was up-regulated more than 14.08-fold in *E*. *coli* cells under 1 MPa treatment. SoxS is a transcription factor, along with SoxR, that activates the expression of several genes involved in superoxide dismutase (SOD) system which is identified as the main defence mechanism that protects the bacteria from the toxic effects of high oxygen saturation [27].

Both Aertsen et al. [11] and Bowman et al. [28] reported that the SOS response which triggers the production of DNA repair proteins following DNA damage was also found to be induced by high pressure. Malone and colleagues found the expression of the *nrdHIEF* operon was up-regulated when *E*. *coli* cells were treated with 100 MPa [3]. The *nrdHIEF* operon encodes a ribonucleotide reductase (RRase) which provides the building blocks for DNA biosynthesis. The over expression of this operon was triggered in response to oxidative stress, particularly in mutants missing major antioxidant defences and in cells treated with oxidants [29]. Malone suggested that an enhanced RRase may protect DNA against reactive oxygen species escaping from the antioxidant defences [3]. Interestingly, we also found this whole operon was upregulated when the *E*. *coli* cells were treated at 1 MPa, which suggests that the SOS response can be triggered at relevantly low elevated pressure.

We also found *grxA*, encoding for a glutaredoxin (Grx1), had a 6.34-fold increase in expression under 1 MPa treatment. This small protein was also found up regulated under 100 MPa treatment along with two thioredoxins (Trx1 and Trx2) [3]. It was reported to be induced by H_2_O_2_ in an OxyR-dependent fashion [30]. OxyR is a transcriptional regulator sensitive to oxidation and activates the expression of antioxidant genes. Although we did not see the changes in OxyR in our study, but we believe that Grx1 play an important role in antioxidant defences and maintaining redox homeostasis when cells are treated with elevated pressure.

We also found up-regulation of several operons are high oxygen level related. The RNA-Seq data shows the up-regulation of CyoA, CyoB and CyoC which are three individual subunits of a cytochrome o oxidase. In the presence of oxygen, *E*. *coli* can respire by using either of two distinct cytochromeoxidases, cytochrome o oxidase (encoded by *cyoABCDE*) and cytochrome d oxidase (encoded by *cydAB* operon). Cotter reported that the expression of these two operons depends on the level of available oxygen, and *cyoABCDE* appears to be produced only under oxygen-rich growth conditions [31]. We also found that *sdhC* and *sdhD* were upregulated when the cells treated with pressure. Succinate dehydrogenase (SDH), encoded by *sdhCDAB*, is the only membrane-bound enzyme involved in TCA cycle, and it is up-regulated by aerobiosis [32]. Interestingly, both *cyo* and *sdh* operons are regulated by ArcA [32, 33]. Although we did not find a change in *acrA* expression, the overexpression of these two operons confirmed that elevated pressure treatment increased the solubility of the oxygen in the system and, to a certain extent, may have induced the ROS and SOS response discussed above.

### Fe-S clusters assembly

Iron-sulfur clusters (Fe-S) containing proteins are crucial in all organisms. They are involved in a broad range of cellular activities including redox and non-redox catalysis. Fe-S clusters exist in various forms including 4Fe–4S clusters, 2Fe–2S clusters and 3Fe–4S clusters, and it often depend on the availability of oxygen [34–36]. In *E*. *coli*, the assembly of Fe-S clusters involves two pathways: housekeeping ISC (iron sulfur cluster) system, and system under stress conditions, the SUF (sulfur formation) system. ISC system encoded by the *iscRSUA-hscBA-fdx-iscX* operon [37–41] and SUF system is encoded by the *sufABCDSE* operon. [42–44]. The SUF system was reported to be active when the cells are under stress conditions such as iron starvation, oxidative damage and heavy metal exposure [43–47]. There are conflicting findings regarding the expression of Fe-S assembling genes when *E*. *coli* cells were under pressure. Malone et al [3] reported that the sublethal pressure (100 MPa) treatment cells down regulated the entire *suf* operon, and they also found the *iscR* was significantly up-regulated under same treatment which represses the *isc* operon. On the other hand, Ishii et al. [10] found several genes involved in *isc* operon which were up regulated when *E*. *coli* cells were treated with 30 MPa and 50 MPa. They include *iscR*, *iscS*, *iscA*, *hscA*, *hscB* and *fdx*. Similarly, we found all the genes in *isc* operon were up regulated at different levels, which indicated that there is a high level of activity of Fe-S clusters of assembly in the cells which are subject to the mild elevated pressure. As discussed before, it is likely that pressure treatment increases the solubility of oxygen which could cause the damage to some proteins containing Fe-S cluster and lead to the destruction of Fe-S clusters and thus release Fe. We propose that under mild elevated pressure, the assembly of Fe-S clusters were encouraged to compensate for the damage caused by the extra oxygen level in the cells. This is also supported by Roche’s finding that under iron rich conditions iron binds to IscX and changes its configuration and modify its interaction with IscS therefore increase the assembly of Fe-S cluster [48].

### Iron homeostasis

Iron is an essential element for most organisms and it plays vital roles in many cellular process including DNA biosynthesis, the TCA cycle, N_2_ fixation and oxygen transport [49]. Iron acquisition in *E*. *coli* is regulated by availability of iron in the cells through 7 transport systems of which 5 exist for Fe ^3+^ and 2 exist for Fe^2+^ [50–53]. In general, *E*. *coli* utilizes siderophores to solubilize extracellular ferric irons (Fe^3+^). The ferric-siderophores complexes will be bound by specific outer membrane receptors and shuttled into the inner membrane through ABC transport system / TonB-ExbB-ExbD system and eventually delivered to the cytosol. In the cytoplasm, iron will be released through the reduction of ferric iron to ferrous iron catalyzed by ferric iron reductases [54]. It is well studied that there are six siderophore receptors produced by *E*. *coli*, which are Cir, FecA, FepA, FhuA, FhuE, and Fiu. It also has three ferri-siderophore periplasmic-binding protein-dependent ABC-transporter systems, FecBCDE, FepBCDEFG, and FhuBCD. *E*. *coli* cells also can take up ferrous iron through EfeUOB and Feo system [55].

The expression of iron metabolism genes including iron acquisition is regulated by ferric uptake regulator (Fur). Under iron- replete condition, Fur will bind with Fe^2+^ cofactor and form holoenzyme (holo Fur). Then it binds to the promoter region of several iron metabolism operons and significantly decreases transcription of those genes within the operons. Seo *et al*. reported more than 60 genes in *E*. *coli* were repressed in this manner [49], and they are including genes involved in 1) the uptake of ferrous irons, *efeUOB* and *feoABC*, 2) synthesis of enterobactin, the most efficient siderophore, *entCEBAH*; 3) ferric siderophore complex receptors and their transport system, such as *fepABCDEG* and *fecIRABCDE*.

Interestingly, our RNA-Seq data has shown a strong evidence that there was up-regulation in iron acquisition system in *E*. *coli* subject to 1 MPa. We have seen that the entire *ent* operon was upregulated nearly 15 fold when the cells were treated with pressure. In line with this change, the receptor for enterobactin-Fe complex FepA and proteins involved in transportation of this complex into cytoplasm FepB, FepC, FepD and FepG were observed to be up-regulated with 13.14 fold, 5.95 fold, 5.61 fold, 3.80 fold and 5.32 fold respectively. Indeed, we found 5 out of 6 well-known siderophore receptors (except FhuA) were up-regulated under pressure treatment to different levels, and so did proteins involved in transport system such as FecA, FecB, FecC, FecD and FecE.

The upregulation of iron acquisition system in *E*. *coli* indicates that there was a decrease in holo Fur (Fur- Fe^2+^) activity. The effect of oxygen level on iron metabolism of *E*. *coli* has been studied intensively in the literature. The soluble reduced iron Fe^2+^ in the presence of oxygen could trigger the Fenton reaction which leads to serious cell damage [56]. Studies show that transcription of *fur* is activated by the OxyR regulator when it gets oxidized by H_2_O_2_ and SoxRS in response to superoxide inducers [57]. In turn the Fur-Fe^2+^ will repress iron-uptake to avoid accumulation of free iron which could lead to Fenton reaction. Therefore, *E*. *coli* has strict and elaborate iron regulation to enable cells to acquire a sufficient amount of iron and avoid causing oxidative stress and damage. However, the lack of iron availability in the presence of oxygen could be a major problem for bacteria cells due to the poor solubility of the oxidized Fe^3+^ form. As we have discussed above there are many indications that the level of the oxygen in the cells was increased when *E*. *coli* was exposed to 1 MPa. The upregulation of the iron-uptake systems found in our experiment seems to suggest that iron was oxidized from Fe^2+^ to Fe^3+^ due to the higher soluble oxygen level induced by the pressure treatment, so the Fur-Fe^2+^ complex becomes its apo format and therefore induced the iron metabolism operons. In addition, Imlay and his group show that *E*. *coli* Hpx^−^ mutants, which lack peroxidase and catalase activities, accumulate H_2_O_2_ intracellularly and the iron-import proteins which Fur normally represses were fully induced in those strains. They suggested that H_2_O_2_ may antagonise Fur function by oxidizing the Fur-Fe^2+^ complex and inactivating its repressor function [58]. In the same vein, as mentioned before, we found the entire *nrdHIEF* operon, which is manganese-containing ribonucleotide reductase complex repressed by Fur-Fe^2+^ [59], was up-regulated. We propose, therefore, that when bacteria cells are treated with mild elevated pressure, the concentration of cellular oxygen increases promptly and subsequently damages the Fur-Fe^2+^ complex and produces apo Fur leading to the increased expression of proteins involved in the iron acquisition.

The differentially expressed genes discovered in this work are based on two biological with two technical replicates analysed using RNA-Seq. Among 101 differentially expressed genes, two up-regulated genes (*azuC* and *entC*) were chosen for validation using RT-qPCR. Due to different normalization methods applied in RNA-Seq and RT-qPCR, a discrepancy was observed in the Fc values for both genes. However, the up-regulation of both genes was proved valid.

These two genes were chosen for further study by constructing translational GFP reporter strains and monitoring their response to pressure. To our surprise, the *entC* translational report strain did not show as significant a signal change under 1 MPa as shown in RNA-Seq and RT-qPCR results. We suspect that there were two potential reasons for this inconsistency: 1) different media were used in those experiments, i.e. LB broth was used in RNA-Seq and RT- qPCR, in contrast, M9 medium was used in fluorescent signal measurement for GFP strains to minimise the background noise; 2) RNA-Seq and RT-qPCR results represent the changes at transcriptional level. In contrast, GFP signal represent the changes at translational level.

More importantly, we observed a linear change in expression of *azuC* in response to the increase of pressure. There is a possibility that the linear change in expression of *azuC* is not directly caused by pressure and more likely related to the increase in the oxygen level as Hemm *et al*. suggested that this gene can be over expressed when the cells are under oxidative stress [19]. Nevertheless, this change indicates the mechanisms that *E*. *coli* use when adapting to elevated pressure of air containing oxygen.

In conclusion, we analysed the gene expression changes for *E*. *coli* under 1 MPa hydrostatic pressure using RNA-Seq. Our results show that mild elevated pressure has an impact on *E*. *coli* cells especially in the iron metabolism system. *E*. *coli* have developed a mechanism to adapt to the changes caused by low level pressure increases. Our study also demonstrated changes in the expression of certain genes, especially stress related genes, which show linear relationships with pressure at very low levels. These findings provide preliminary data for creating pressure-sensing strains, which potentially have broad applications such as detecting mechanical changes in soil substrates for soil improvement. In addition, the majority of differentially expressed genes in our study are up-regulated. This could be due to the limited biological replicates we used in RNA-Seq [60]. Further work is needed to increase the statistical power and detect more differentially expressed genes by using more biological replicates in both control and pressured conditions.

## Supporting information

S1 Fig Photographs of pressure vessel for pressure treatment setup. A. 10 bar regulator; B. Synthetic air bottle filled to 300 bar; C. Pressure vessel; D. Digital thermometer; E. Temperature sensor; F. Input valve; G. Pressure gauge; H. Output valve; J. Rubber sealing ‘O’ ring; K. Eppendorf's mounted on foam; L. 1 mm hole drilled into the Eppendorf lid.
(TIF)

S2 Fig pAGcc3 and pAGcc4 plasmid maps (SnapGene viewer design). These plasmids were engineered by Gibson assembly cloning and were used as PCR matrix to integrate the GFP-reporter system to a specific locus of the *E*. *coli* MG1655 chromosome using the λ Red recombinase method. NEBuilder, Benchling and SnapGene viewer softwares were used to design the cloning, primers and the final plasmid maps. The origins of the assembled parts are represented in the outside circle on the maps. *cat*: chloramphenicol. (A) Plasmid pAGcc3 carries a translational fusion of *azuC* to gene encoding monomeric superfolder-GFP and the flanking regions of *azuC* locus to allow double cross-over to *E*. *coli* chromosome. (B) Plasmid pAGcc4 carries a 3’-terminus DNA part of *entC* coupled in translational fusion to the gene encoding the monomeric superfolder-GFP. Plasmid carries the intergenic region of promoter *PentC* to allow transcription of the operon *entEBAH* after recombination at the *entC* chromosome locus.
(TIF)

S3 Fig Temperature change during the periods of pressure treatment for RNA-Seq experiment. Temperatures within pressure vessel were recorded during the pressurization process.
(TIF)

S4 Fig PCA plot of the global gene expression of *E*. *coli* samples analysed by RNA-seq. The eight samples from two biological replicates, each with two technical replicates for each condition were analysed. HP samples were treated with high pressure, i.e. 1 MPa; LPs were control samples.
(TIF)

S1 Table Plasmids and strains used in this study. *lacZ*, *β*-galactosidase gene; *neo*, neomycin resistance gene; T0, terminator of transcription; *bla*, ampicillin resistance gene, *P*_*araB*_, arabinose-inducible promoter; *cat*, chloramphenicol resistance gene; *spc*, spectinomycin resistant gene; *P*, promoter. *msfgfp*, gene encoding the monomeric superfolder green fluorescent protein.
(DOCX)

S2 Table Genes studied by qPCR and the sequences of primers. ^a^Amplicon size expected by PCR when using the indicated primers pair. ^b^Housekeeping gene. ^c^Targeted—pressure sensitive genes.
(DOCX)

S3 Table Primers used in translational fusion construction. GC: Gibson assembly cloning; CDS: coding DNA sequence; msfGFP: monomeric superfolder green fluorescent protein; *cat*: chloramphenicol.
(DOCX)

S4 Table Up-regulated *E*. *coli* genes in response to 1 MPa treatment. ^a^EcoCYC accession ID.^b^COG categories: [C]—Energy production and conversion; [E] -Amino acid transport and metabolism; [F]—Nucleotide transport and metabolism; [G]—Carbohydrate transport and metabolism; [H]—Coenzyme transport and metabolism; [I]—Lipid transport and metabolism; [J]—Translation, ribosomal structure and biogenesis; [K]—Transcription; [M]—Cell wall/membrane/envelope biogenesis; [O]–Post-translational modification, protein turnover, chaperones; [P]- Inorganic ion transport and metabolism; [Q]—Secondary metabolites biosynthesis, transport and catabolism; [R]–General function prediction only; [S]—Function unknown [T]—Signal transduction mechanisms and [no info]—no information was available for this gene at the time of this study.
(DOCX)

S5 Table Down-regulated *E*. *coli* genes in response to 1MPa treatment. ^a^EcoCYC accession ID. ^b^COG categories: [C]—Energy production and conversion; [H]—Coenzyme transport and metabolism; [K]–Transcription; [O]–Post-translational modification, protein turnover, chaperones; [P]- Inorganic ion transport and metabolism; [R]–General function prediction only; [S]—Function unknown and [no info]—no information was available for this gene at the time of this study.
(DOCX)

S1 File Raw data for RT-qPCR experiments. Raw CT values obtained for one biological sample tested in triplicate (technical replicate) by RT-qPCR. RT-qPCR reactions were performed on gene *rrsA* (reference gene), *azuC* and *entC* of *E*. *coli*. The high-pressure samples (HP) refer to the tubes with modified caps with 1 mm holes drilled through the top, and the low-pressure samples (LP) refer to the tubes sealed with normal caps to protect them from pressure change. Both sets of samples were place inside the pressure vessel. No pressure samples refer to samples were placed outside the pressure vessel.
(XLSX)

S2 File Raw data for strains response to different pressure stress. The TECAN Spark^TM^ 10M multimode Microplate reader was used to monitor the optical cell density at OD_600 nm_ and the GFP signal with an excitation 485 nm /emission filter 510 nm. *E*. *coli* strain MG1655 (MG), HS524 (HS) and AG1319 (AG) were negative control, positive control and testing strain respectively. M9 medium was used as a blank condition (BL). The samples, which were pressurised in the pressure vessel, were labelled as IN and the samples which were placed outside the pressure vessel were labelled as OUT. Each assay contained three biological samples, with technical triplicates for each biological sample.
(XLSX)
