# Supplementary figures and images for: Mild hydrostatic pressure triggers oxidative responses in Escherichia coli
Source: PLoS One. 2018 Jul 17;13(7):e0200660. doi: 10.1371/journal.pone.0200660 (PMC6049941; doi:10.1371/journal.pone.0200660)

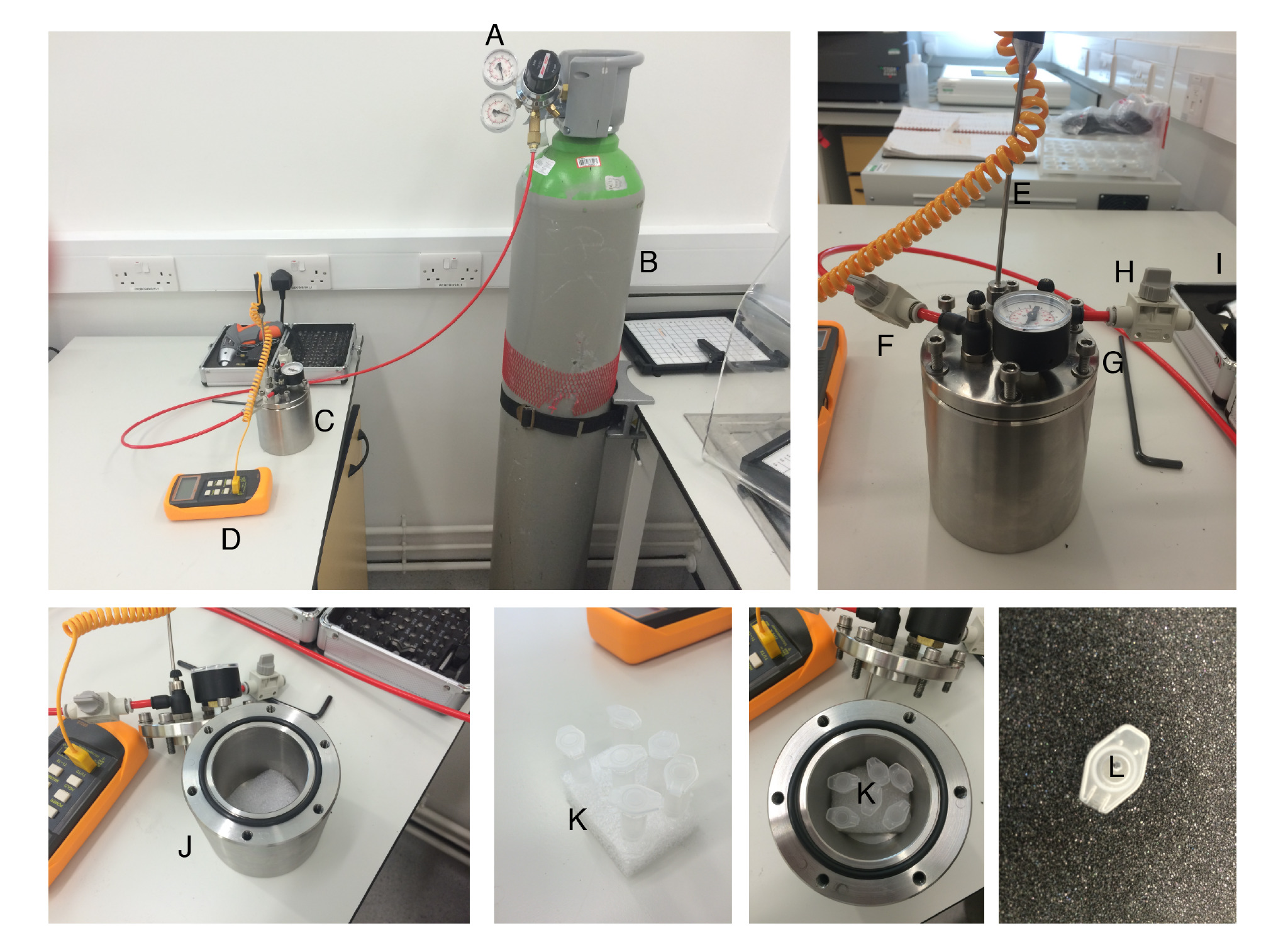

Supplement: S1 Fig — A. 10 bar regulator; B. Synthetic air bottle filled to 300 bar; C. Pressure vessel; D. Digital thermometer; E. Temperature sensor; F. Input valve; G. Pressure gauge; H. Output valve; J. Rubber sealing ‘O’ ring; K. Eppendorf's mounted on foam; L. 1 mm hole drilled into the Eppendorf lid. (TIF) [file pone.0200660.s001.tif]

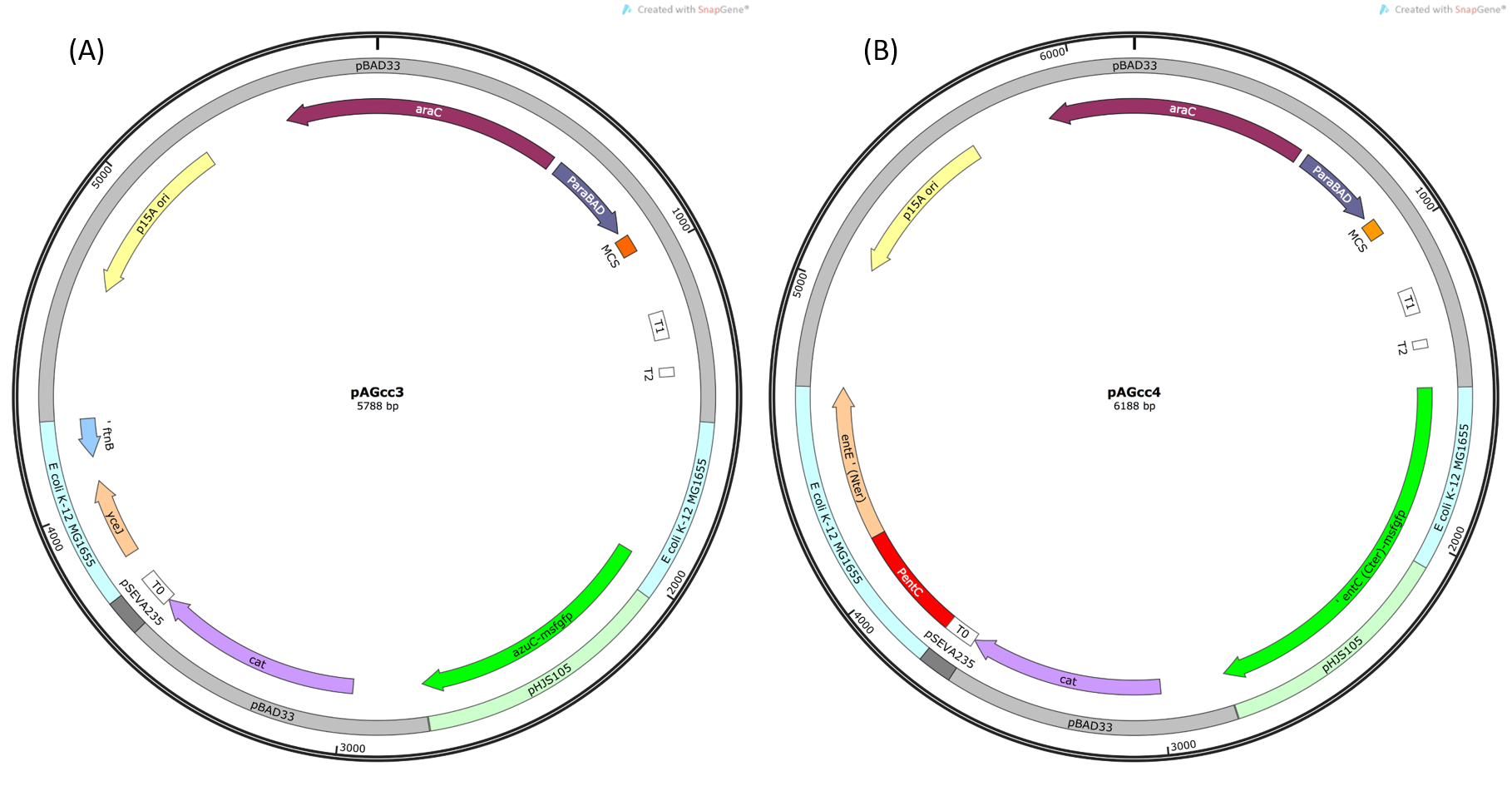

Supplement: S2 Fig — These plasmids were engineered by Gibson assembly cloning and were used as PCR matrix to integrate the GFP-reporter system to a specific locus of the E. coli MG1655 chromosome using the λ Red recombinase method. NEBuilder, Benchling and SnapGene viewer softwares were used to design the cloning, primers and the final plasmid maps. The origins of the assembled parts are represented in the outside circle on the maps. cat: chloramphenicol. (A) Plasmid pAGcc3 carries a translational fusion of azuC to gene encoding monomeric superfolder-GFP and the flanking regions of azuC locus to allow double cross-over to E. coli chromosome. (B) Plasmid pAGcc4 carries a 3’-terminus DNA part of entC coupled in translational fusion to the gene encoding the monomeric superfolder-GFP. Plasmid carries the intergenic region of promoter PentC to allow transcription of the operon entEBAH after recombination at the entC chromosome locus. (TIF) [file pone.0200660.s002.tif]

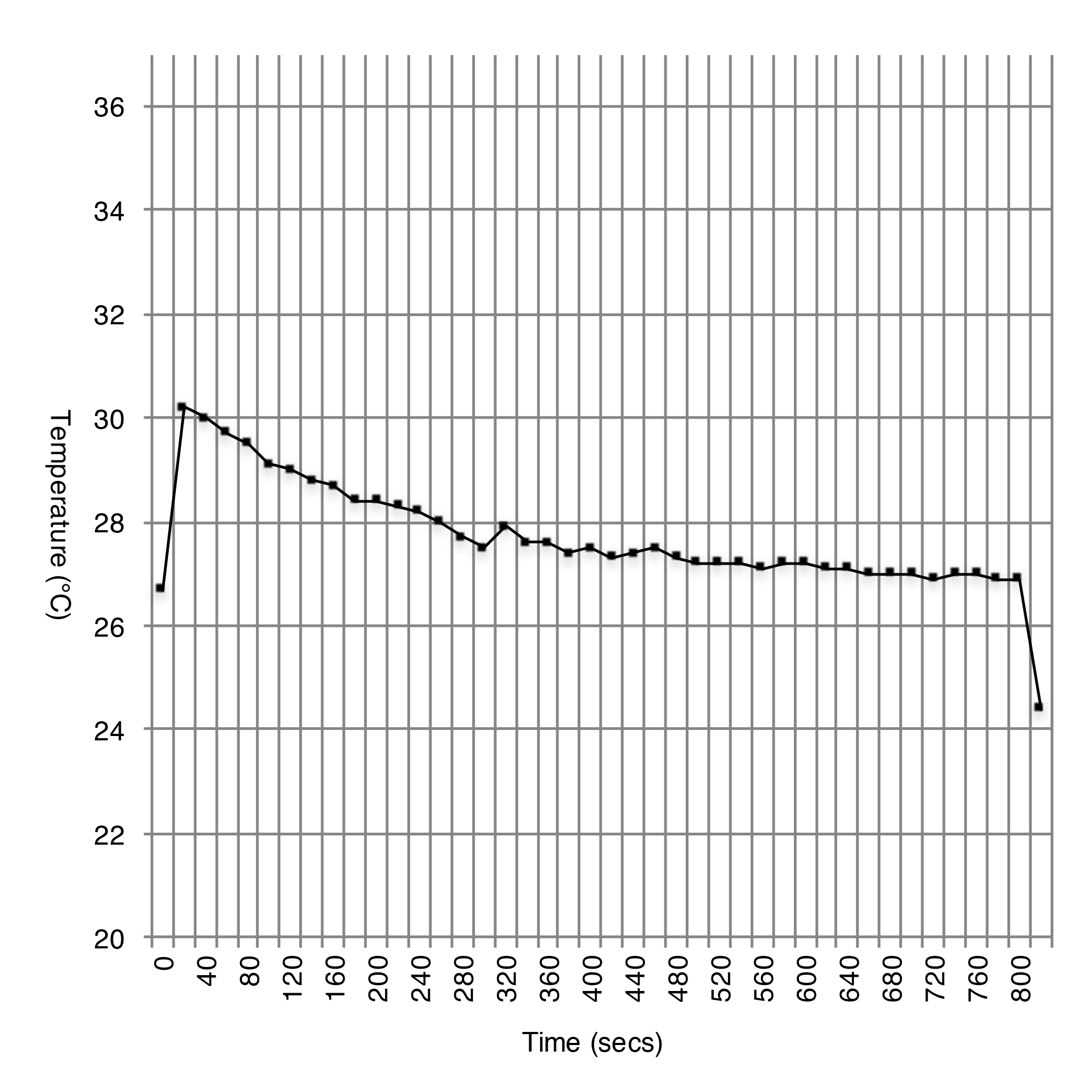

Supplement: S3 Fig — Temperatures within pressure vessel were recorded during the pressurization process. (TIF) [file pone.0200660.s003.tif]

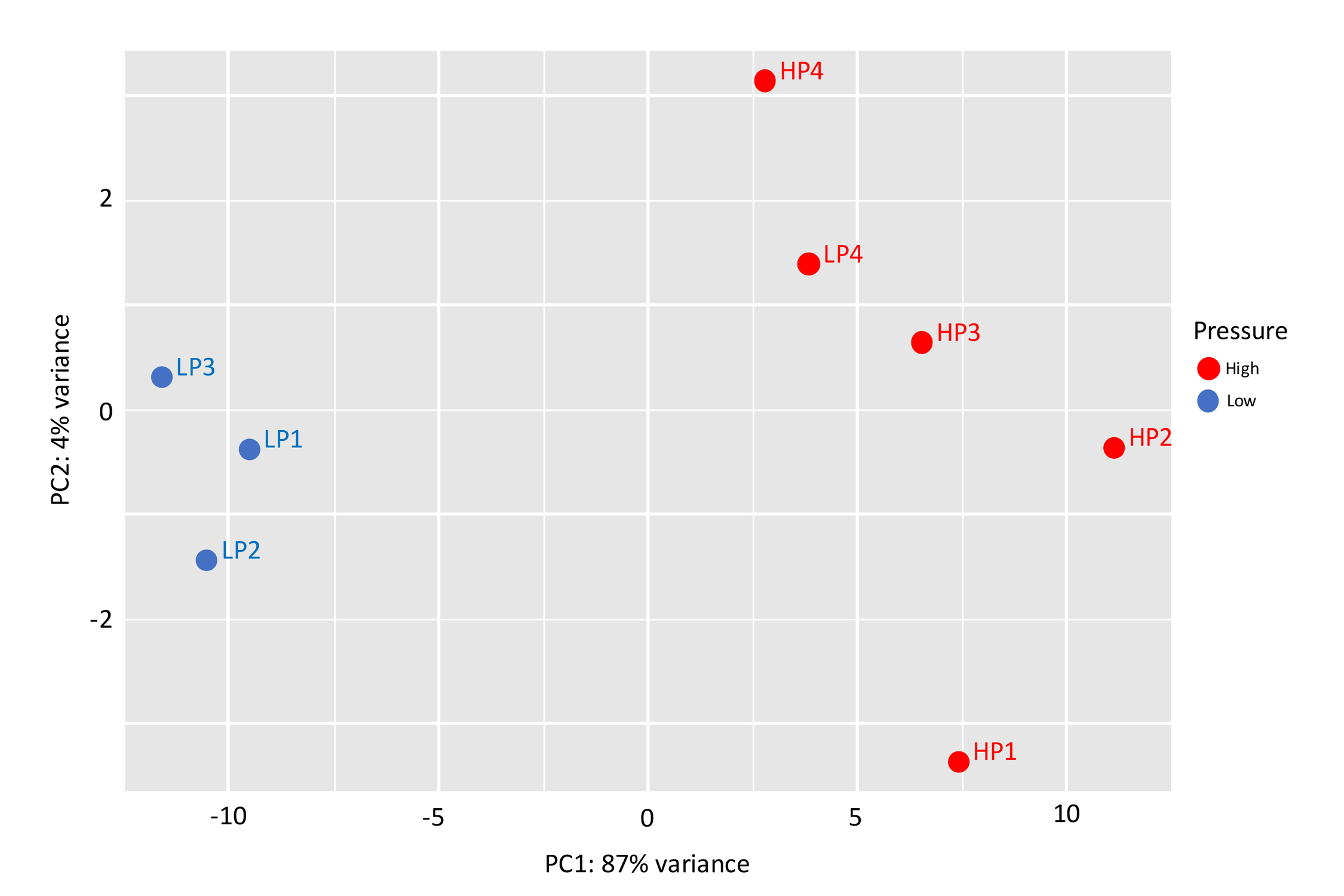

Supplement: S4 Fig — The eight samples from two biological replicates, each with two technical replicates for each condition were analysed. HP samples were treated with high pressure, i.e. 1 MPa; LPs were control samples. (TIF) [file pone.0200660.s004.tif]
