# Supplementary material for: Mild hydrostatic pressure triggers oxidative responses in Escherichia coli
Source: PLoS One. 2018 Jul 17;13(7):e0200660. doi: 10.1371/journal.pone.0200660 (PMC6049941; doi:10.1371/journal.pone.0200660)
Supplement: S1 Table — lacZ, β-galactosidase gene; neo, neomycin resistance gene; T0, terminator of transcription; bla, ampicillin resistance gene, ParaB, arabinose-inducible promoter; cat, chloramphenicol resistance gene; spc, spectinomycin resistant gene; P, promoter. msfgfp, gene encoding the monomeric superfolder green fluorescent protein. (DOCX) [file pone.0200660.s005.docx]

**S1 Table. Plasmids and strains used in this study.**

| Name | Key features | References |
| --- | --- | --- |
| Plasmids |  |  |
| pSEVA235 | *lacZ*, *neo,* T0 | [1] |
| pBAD33 | *cat*, p15A origin | [2] |
| pKD46 | *bla,* *P_araB_*, *gam bet exo* (lambda Red genes), repA101ts (grow at 30°C) | [3] |
| pKD3 | *Bla, cat* | [3] |
| pHJS105 | *bla, amyE3', spc, Pxyl-msfgfp, amyE5'* | [4] |
| pAGcc3 | derivative pBAD33, carry *PazuC-azuC-msfgfp*, *cat*, T0, and flanking DNA regions of *azuC* | This study |
| pAGcc4 | derivative pBAD33, carry *PentC-entC-msfgfp*, *cat*, T0, *PentC* and flanking DNA regions of *entC* locus | This study |
| *E. coli* strains |  |  |
| K-12 MG1655 | *F-, lambda-, rph-1* | Wipat lab collection |
| MG1655 pKD46 | pKD46 | Wipat lab collection |
| NEB 5α | *fhuA2 Δ(argF-lacZ)U169 phoA glnV44 Φ80 Δ(lacZ)M15 gyrA96 recA1 relA1 endA1 thi-1 hsdR17* | NEB, E5510S kit |
| AG1319 | MG1655 *P*_azuC_-*azuC-msfgfp*, *cat* | This study |
| AG1321 | MG1655 *P_entC_-entC-msfgfp*, *cat*, *P_entC_*-*entEBAH* | This study |
| HS524 | MG1655 *mreB-msfgfp* | [5] |

*lacZ,* β-galactosidase gene; *neo,* neomycin resistance gene; T0, terminator of transcription; *bla*, ampicillin resistance gene, *P_araB_*, arabinose-inducible promoter; *cat*, chloramphenicol resistance gene; *spc*, spectinomycin resistant gene; *P,* promoter. *msfgfp*, gene encoding the monomeric superfolder green fluorescent protein.

**References**

1. Silva-Rocha R, Martinez-Garcia E, Calles B, Chavarria M, Arce-Rodriguez A, de Las Heras A, et al. The Standard European Vector Architecture (SEVA): a coherent platform for the analysis and deployment of complex prokaryotic phenotypes. Nucleic Acids Res. 2013;41(Database issue):D666-75. doi: 10.1093/nar/gks1119. PubMed PMID: 23180763; PubMed Central PMCID: PMCPMC3531073.

2. Guzman LM, Belin D, Carson MJ, Beckwith J. Tight regulation, modulation, and high-level expression by vectors containing the arabinose PBAD promoter. J Bacteriol. 1995;177(14):4121-30. PubMed PMID: 7608087; PubMed Central PMCID: PMCPMC177145.

3. Datsenko KA, Wanner BL. One-step inactivation of chromosomal genes in *Escherichia coli* K-12 using PCR products. P Natl Acad Sci USA. 2000;97(12):6640-5. PubMed PMID: WOS:000087526300074.

4. Jahn N, Brantl S, Strahl H. Against the mainstream: the membrane-associated type I toxin BsrG from *Bacillus subtilis* interferes with cell envelope biosynthesis without increasing membrane permeability. Mol Microbiol. 2015;98(4):651-66. Epub 2015/08/04. doi: 10.1111/mmi.13146. PubMed PMID: 26234942.

5. Ouzounov N, Nguyen JP, Bratton BP, Jacobowitz D, Gitai Z, Shaevitz JW. MreB Orientation Correlates with Cell Diameter in *Escherichia coli*. Biophys J. 2016;111(5):1035-43. Epub 2016/09/08. doi: 10.1016/j.bpj.2016.07.017. PubMed PMID: 27602731; PubMed Central PMCID: PMCPMC5018124.
