# Supplementary material for: Mild hydrostatic pressure triggers oxidative responses in Escherichia coli
Source: PLoS One. 2018 Jul 17;13(7):e0200660. doi: 10.1371/journal.pone.0200660 (PMC6049941; doi:10.1371/journal.pone.0200660)
Supplement: S2 Table — aAmplicon size expected by PCR when using the indicated primers pair. bHousekeeping gene. cTargeted—pressure sensitive genes. (DOCX) [file pone.0200660.s006.docx]

**S2 Table. Genes studied by qPCR and the sequences of primers.**

| **Gene** | **Description** | **Primer sequence 5' to 3'** | | **Amplicon size (bp)^a^** |
| --- | --- | --- | --- | --- |
| *rrsA*^b^ | Ribosomal RNA, 16S | F | CGCATCATGCAGTATTCCAG | 124 |
|  |  | R | CACAATATCAGCCCCCAATG |  |
| *azuC*^c^ | Acid-inducible small membrane-associated protein | F | ATGAAACTGCGCAAAATCC | 70 |
|  |  | R | CTGGCGGTACGTCTTTGA |  |
| *entC*^c^ | Isochorismate synthase 1 | F | CTCCCGTCAGGAAAAACAAG | 92 |
|  |  | R | TGGTTTGCTCCGGAATTG |  |

^a^Amplicon size expected by PCR when using the indicated primers pair. ^b^Housekeeping gene. ^c^Targeted - pressure sensitive genes.
