# Supplementary material for: Mild hydrostatic pressure triggers oxidative responses in Escherichia coli
Source: PLoS One. 2018 Jul 17;13(7):e0200660. doi: 10.1371/journal.pone.0200660 (PMC6049941; doi:10.1371/journal.pone.0200660)
Supplement: S3 Table — GC: Gibson assembly cloning; CDS: coding DNA sequence; msfGFP: monomeric superfolder green fluorescent protein; cat: chloramphenicol. (DOCX) [file pone.0200660.s007.docx]

**S3 Table. Primers used in translational fusion construction.**

| **Primer name** | **sequence** | **Source Matrix used** | **Aim^a^** |
| --- | --- | --- | --- |
| **AG649** | GTCGGGTGATGCTGCCAACTTAC | pBAD33 | GC, amplify pBAD33 partial |
| **AG650** | GACACGGAAATGTTGAATACTC | pBAD33 | GC, amplify pBAD33 partial |
| **AG651** | aaaggaagagtatgagtattcaacatttccgtgtcGCGATCCCGCGACTGACATA | MG1655 | GC, amplify DNA upstream *azuC* and *azuC*-CDS without stop codon |
| **AG652** | acaactccagtgaaaagttcttctcctttgctcatTCGGAACATATTGCCTGGCG | MG1655 | GC, amplify DNA upstream *azuC* and *azuC*-CDS without stop codon |
| **AG653** | ATGAGCAAAGGAGAAGAACTTTTC | pHJS105 | GC, amplify msfGFP-CDS with stop codon |
| **AG654** | ttcggcgcaaagtgcttaTTTGTAGAGCTCATCCATGC | pHJS105 | GC, amplify msfGFP-CDS with stop codon |
| **AG655** | atggatgagctctacaaataaGCACTTTGCGCCGAATAAATACCTGTGACG | pBAD33 | GC, amplify cat resistance cassette (promoter and CDS) |
| **AG656** | tatcaacaggagtccaagTTACGCCCCGCCCTGCCACT | pBAD33 | GC, amplify cat resistance cassette (promoter and CDS) |
| **AG657** | tggcagggcggggcgtaaCTTGGACTCCTGTTGATAGATC | pSEVA235 | GC, amplify terminator T0 |
| **AG658** | CTGGATTCTCACCAATAAAAAAC | pSEVA235 | GC, amplify terminator T0 |
| **AG659** | ttgccgccgggcgttttttattggtgagaatccagGTGTCCTGATGACGGTGGAT | MG1655 | GC, amplify DNA downstream *azuC* for recombination |
| **AG660** | tacactaaatcagtaagttggcagcatcacccgacCAGCATGATGGTCTGTTGCTG | MG1655 | GC, amplify DNA downstream *azuC* for recombination |
| **AG661** | aaaggaagagtatgagtattcaacatttccgtgtcGCTCCATTCCGTTAGCCGGT | MG1655 | GC, amplify 3’ terminal part of *entC*-CDS without the stop codon |
| **AG662** | acaactccagtgaaaagttcttctcctttgctcatATGCAATCCAAAAACGTTCAACATGG | MG1655 | GC, amplify 3’ terminal part of *entC*-CDS without the stop codon |
| **AG663** | ttgccgccgggcgttttttattggtgagaatccagGGCGAGTCTCACAAATCAGC | MG1655 | GC, amplify promoter of *entC* |
| **AG664** | ggtgaatggaatgctcatATCATCCTCCACAAAATGATAAAGG | MG1655 | GC, amplify promoter of *entC* |
| **AG665** | cattttgtggaggatgatATGAGCATTCCATTCACCCG | MG1655 | GC, amplify downstream *entC* for recombination |
| **AG666** | tacactaaatcagtaagttggcagcatcacccgacTCCTCAGCCGGATGGTTAAT | MG1655 | GC, amplify downstream *entC* for recombination |
| **AG670** | tccgctcatgagacaataacc | pBAD33 pAGcc3-4 | bind pBAD33, to check assembly part in GC |
| **AG671** | gcacctcaaaaacaccatca | pBAD33 pAGcc3-4 | bind pBAD33, to check assembly parts in GC |
| **AG675** | ATTGTGGGTTGGTGTGACAG | MG1655 | use with AG676, to check mutation locus *entC* |
| **AG676** | CCGATAACTCAACTGTCGCT | MG1655 | use with AG675, to check mutation locus *entC* |
| **AG677** | TGCCATCTGTAGGTGTCAGA | MG1655 | use with AG678, to check mutation locus *azuC* |
| **AG678** | TGATAACCAACTGGCAAGCA | MG1655 | use with AG677, to check mutation locus *azuC* |

^a^GC: Gibson assembly cloning; CDS: coding DNA sequence; msfGFP: monomeric superfolder green fluorescent protein; *cat*: chloramphenicol.
