# Supplementary material for: Mild hydrostatic pressure triggers oxidative responses in Escherichia coli
Source: PLoS One. 2018 Jul 17;13(7):e0200660. doi: 10.1371/journal.pone.0200660 (PMC6049941; doi:10.1371/journal.pone.0200660)
Supplement: S4 Table — aEcoCYC accession ID.bCOG categories: [C]—Energy production and conversion; [E] -Amino acid transport and metabolism; [F]—Nucleotide transport and metabolism; [G]—Carbohydrate transport and metabolism; [H]—Coenzyme transport and metabolism; [I]—Lipid transport and metabolism; [J]—Translation, ribosomal structure and biogenesis; [K]—Transcription; [M]—Cell wall/membrane/envelope biogenesis; [O]–Post-translational modification, protein turnover, chaperones; [P]- Inorganic ion transport and metabolism; [Q]—Secondary metabolites biosynthesis, transport and catabolism; [R]–General function prediction only; [S]—Function unknown [T]—Signal transduction mechanisms and [no info]—no information was available for this gene at the time of this study. (DOCX) [file pone.0200660.s008.docx]

**S4 Table. Up-regulated *E. coli* genes in response to 1MPa treatment.**

| **Gene** **Name** | **Gene ID^a^** | **Description** | **log2Fold** | **Fc** | **COG^b^** |
| --- | --- | --- | --- | --- | --- |
|  |  |  | **Change** |  |  |
| *azuC* | G0-10653 | small membrane protein | 2.61 | 3.04 | no info |
| *betT* | EG10112 | choline:H^+^ symporter | 2.17 | 8.79 | M |
| *bioA* | EG10117 | adenosylmethionine-8-amino-7-oxononanoate aminotransferase | 1.61 | 7.29 | H |
| *bluF* | G6603 | blue light-responsive regulator of BluR | 2.59 | 9.58 | T |
| *cirA* | EG10155 | ferric dihyroxybenzoylserine outer membrane transporter | 5.37 | 21.02 | P |
| *cyoA* | EG10178 | cytochrome *bo_3_* terminal oxidase subunit II | 2.09 | 10.23 | C |
| *cyoB* | EG10179 | cytochrome bo terminal oxidase subunit I | 1.84 | 9.81 | C |
| *cyoC* | EG10180 | cytochrome bo terminal oxidase subunit III | 1.69 | 8.48 | C |
| *efeB* | EG11735 | heme-containing peroxidase/deferrochelatase | 2.43 | 6.4 | P |
| *efeO* | G6527 | periplasmic protein, component of cryptic ferrous ion transporter | 3.27 | 8.44 | P |
| *entA* | EG10259 | 2,3-dihydro-2,3-dihydroxybenzoate dehydrogenase | 3.33 | 10.28 | IQR |
| *entB* | EG10260 | holo EntB, EntB | 5.19 | 13.58 | Q |
| *entC* | EG10261 | isochorismate synthase 1 | 5.46 | 12.71 | HQ |
| *entD* | EG10262 | enterobactin synthetase component D, enterochelin synthase D | 2.34 | 3.76 | Q |
| *entE* | EG10263 | 2,3-dihydroxybenzoate-AMP ligase | 5.08 | 14.77 | Q |
| *entF* | EG10264 | holo [EntF peptidyl-carrier protein], apo-serine activating enzyme | 3.77 | 13.72 | Q |
| *entH* | EG11105 | proofreading thioesterase in enterobactin biosynthesis | 3.69 | 9.49 | Q |
| *entS* | EG11104 | YBDA-MONOMER | 3.1 | 8.19 | GEPR |
| *fdx* | EG11328 | reduced ferredoxin | 2.15 | 11.64 | C |
| *fecA* | EG10286 | ferric citrate outer membrane porin FecA | 2.26 | 13.16 | E |
| *fecB* | EG10287 | ferric citrate ABC transporter - periplasmic binding protein | 2.22 | 12.75 | P |
| *fecC* | EG10288 | ferric citrate ABC transporter - membrane subunit | 2.16 | 9.33 | P |
| *fecD* | EG10289 | ferric citrate ABC transporter - membrane subunit | 1.65 | 7 | P |
| *fecE* | EG10290 | ferric citrate ABC transporter - ATP binding subunit | 1.63 | 6.35 | PH |
| *fecI* | EG10291 | RNA polymerase, sigma 19 factor | 2.26 | 4.24 | K |
| *fecR* | EG10292 | regulator for fec operon, periplasmic | 1.61 | 4.22 | PT |
| *fepA* | EG10293 | ferric enterobactin / colicin B / colicin D outer membrane porin FepA | 4.26 | 13.14 | P |
| *fepB* | EG10294 | ferric enterobactin ABC transporter - periplasmic binding protein | 2.18 | 5.95 | P |
| *fepC* | EG10295 | ferric enterobactin ABC transporter - ATP binding subunit | 2.78 | 5.61 | PH |
| *fepD* | EG10296 | ferric enterobactin ABC transporter - membrane subunit | 2.37 | 3.8 | P |
| *fepG* | EG10298 | ferric enterobactin ABC transporter - membrane subunit | 2.75 | 5.32 | P |
| *fes* | EG10299 | enterochelin esterase | 7.1 | 5.84 | P |
| *fhuE* | EG10306 | ferric coprogen outer membrane receptor FhuE | 2.51 | 8.31 | P |
| *fhuF* | G7949 | hydroxamate siderophore iron reductase | 3.23 | 7.82 | R |
| *fiu* | G6414 | putative outer membrane receptor for iron transport | 5.03 | 19.09 | P |
| *gcd* | EG10369 | quinoprotein glucose dehydrogenase | 1.94 | 12.69 | G |
| *grxA* | EG10417 | reduced glutaredoxin 1 | 2.18 | 6.34 | O |
| *hisM* | EG10007 | lysine/arginine/ornithine ABC transporter / histidine ABC transporter - membrane subunit | 1.78 | 4.55 | E |
| *hscA* | EG12130 | chaperone, member of Hsp70 protein family, chaperone for [Fe-S] cluster biosynthesis | 1.92 | 12.31 | O |
| *hscB* | EG12131 | co-chaperone for [Fe-S] cluster biosynthesis | 2.54 | 15.49 | O |
| *insJ* | G7776 | IS150 protein InsA | 1.64 | 3.43 | no info |
| *insK* | G7777 | IS150 conserved protein InsB | 1.66 | 3.32 | no info |
| *iscA* | EG12132 | iron-sulfur cluster assembly protein | 1.84 | 10.93 | S |
| *iscR* | G7326 | IscR DNA-binding transcriptional dual regulator | 2.69 | 12.64 | K |
| *iscS* | G7325 | cysteine desulfurase | 2.27 | 12.9 | E |
| *iscU* | G7324 | scaffold protein for iron-sulfur cluster assembly | 1.98 | 10.62 | C |
| *iscX* | EG12311 | regulator of iron-sulfur cluster assembly | 1.69 | 9.63 | S |
| *lldP* | EG11961 | (R)-lactate / (S)-lactate / glycolate:H+ symporter LldP | 2.52 | 3.64 | C |
| *lldR* | EG11962 | LldR transcriptional dual regulator | 2.47 | 4.18 | K |
| *metK* | EG10589 | methionine adenosyltransferase | 1.68 | 11.95 | H |
| *mqo* | EG12069 | EG12069-MONOMER | 2.67 | 10.43 | R |
| *ndk* | EG10650 | nucleoside diphosphate kinase | 2.32 | 8.51 | F |
| *nrdE* | EG20257 | ribonucleoside-diphosphate reductase 2, &alpha; subunit | 4.11 | 12.93 | F |
| *nrdF* | EG12381 | ribonucleoside-diphosphate reductase 2, &beta; subunit | 4.41 | 9.26 | F |
| *nrdH* | G7401 | glutaredoxin-like protein | 5.63 | 5.2 | O |
| *nrdI* | G7402 | flavodoxin involved in dimanganese-tyrosyl radical cofactor maintenance for ribonucleotide reductase | 4.82 | 7.04 | F |
| *plaP* | EG11896 | putrescine:H^+^ symporter PlaP | 1.87 | 13.18 | E |
| *potF* | EG11629 | putrescine ABC transporter - periplasmic binding protein | 1.66 | 4.43 | E |
| *pqqL* | EG11744 | putative zinc peptidase | 1.73 | 5.38 | R |
| *purE* | EG10793 | N5-carboxyaminoimidazole ribonucleotide mutase | 1.73 | 7.27 | F |
| *rpsJ* | EG10909 | 30S ribosomal subunit protein S10 | 1.63 | 12.21 | J |
| *rsxD* | G6874 | integral membrane protein of SoxR-reducing complex | 1.63 | 6.18 | no info |
| *sdhA* | EG10931 | succinate:quinone oxidoreductase, FAD binding protein | 1.63 | 3.87 | C |
| *sdhC* | EG10933 | succinate:quinone oxidoreductase, membrane protein SdhC | 2.18 | 4.65 | C |
| *sdhD* | EG10934 | succinate:quinone oxidoreductase, membrane protein SdhD | 2.03 | 4.12 | C |
| *soxS* | EG10958 | SoxS DNA-binding transcriptional dual regulator | 3.69 | 14.08 | K |
| *suhB* | EG10983 | inositol monophosphatase | 2.09 | 12.53 | G |
| *xanP* | EG11194 | xanthine:H^+^ symporter XanP | 1.75 | 7.14 | F |
| *ybdZ* | G0-10437 | MbtH-like protein that enhances the catalytic function of EntF | 4.13 | 3.54 | no info |
| *ybiX* | G6413 | conserved protein | 2.3 | 6.98 | S |
| *yceA* | EG11116 | conserved protein | 2.91 | 12.06 | R |
| *ycfZ* | G6578 | predicted inner membrane protein | 1.98 | 3.85 | no info |
| *ydcI* | G6737 | YdcI DNA-binding transcriptional repressor | 2.18 | 3.54 | K |
| *yddB* | EG11743 | putative TonB-dependent receptor | 2.25 | 4.36 | no info |
| *ydgI* | G6861 | predicted inner membrane transport protein | 1.8 | 8.41 | E |
| *yegQ* | G7118 | predicted peptidase | 1.74 | 9.3 | O |
| *yidD* | G0-10470 | inner membrane protein insertion factor | 1.6 | 9.33 | S |
| *yigI* | EG11467 | putative thioesterase | 1.85 | 4.45 | Q |
| *yjhV* | G7907 | KpLE2 phage-like element; predicted protein | 1.89 | 3.4 | no info |
| *yjjZ* | G0-10474 | putative protein | 3.43 | 3.39 | no info |
| *yncD* | G6762 | putative TonB-dependent outer membrane receptor | 2.64 | 11.47 | P |
| *yncE* | G6763 | conserved protein | 2.87 | 12.18 | S |
| *yoel* | G0-10643 | uncharacterized protein | 2.17 | 3.72 | no info |
| *yojI* | EG12070 | microcin J25 efflux protein, putative ABC superfamily: membrane component / ATP-binding component | 1.75 | 6.14 | QP |
| *yrbN* | G0-10651 | Uncharacterised small protein | 1.86 | 7.64 | no info |

^a^EcoCYC accession ID.^b^COG categories: [C] - Energy production and conversion; [E] -Amino acid transport and metabolism; [F] - Nucleotide transport and metabolism; [G] - Carbohydrate transport and metabolism; [H] - Coenzyme transport and metabolism; [I] - Lipid transport and metabolism; [J] - Translation, ribosomal structure and biogenesis; [K] - Transcription; [M] - Cell wall/membrane/envelope biogenesis; [O] – Post-translational modification, protein turnover, chaperones; [P]- Inorganic ion transport and metabolism; [Q] - Secondary metabolites biosynthesis, transport and catabolism; [R] – General function prediction only; [S] - Function unknown [T] - Signal transduction mechanisms; and [no info] - no information was available for this gene at the time of this study.
