# Supplementary material for: Mild hydrostatic pressure triggers oxidative responses in Escherichia coli
Source: PLoS One. 2018 Jul 17;13(7):e0200660. doi: 10.1371/journal.pone.0200660 (PMC6049941; doi:10.1371/journal.pone.0200660)
Supplement: S5 Table — aEcoCYC accession ID. bCOG categories: [C]—Energy production and conversion; [H]—Coenzyme transport and metabolism; [K]–Transcription; [O]–Post-translational modification, protein turnover, chaperones; [P]- Inorganic ion transport and metabolism; [R]–General function prediction only; [S]—Function unknown and [no info]—no information was available for this gene at the time of this study. (DOCX) [file pone.0200660.s009.docx]

**S5 Table. Down-regulated *E. coli* genes in response to 1MPa treatment.**

| **Gene** **Name** | **Gene ID^a^** | **Description** | **log2Fold** | **Fc** | **COG^b^** |
| --- | --- | --- | --- | --- | --- |
|  |  |  | **Change** |  |  |
| *abrB* | G6384 | predicted regulator | -1.89 | 7.51 | R |
| *bssR* | G6436 | regulator of biofilm formation | -1.72 | 6.68 | no info |
| *Hcp* | G6457 | nitric oxide reductase | -2.17 | 3.45 | C |
| *tdcA* | EG10989 | TdcA DNA-binding transcriptional activator | -16.3 | 3.99 | K |
| *tqsA* | G6859 | autoinducer 2 exporter | -1.67 | 5.6 | no info |
| *ttdR* | EG12694 | Dan transcriptional activator, Dan transcriptional activator | -2.09 | 6.24 | K |
| *ybiY* | G6427 | predicted pyruvate formate lyase activating enzyme | -1.8 | 3.26 | O |
| *ycbJ* | G6473 | conserved protein | -1.9 | 7.26 | no info |
| *yccM* | G6513 | predicted 4Fe-4S membrane protein | -2.54 | 9.5 | C |
| *ydhY* | G6902 | predicted 4Fe-4S ferredoxin-type protein | -1.62 | 5.64 | C |
| *yfbS* | G7186 | putative transport protein | -1.63 | 7.96 | P |
| *yhcC* | G7669 | predicted Fe-S oxidoreductase | -1.76 | 6.79 | R |
| *yidF* | EG11694 | predicted DNA-binding transcriptional regulator | -1.6 | 5.84 | R |
| *yjfN* | G7851 | protease activator | -1.6 | 3.81 | no info |
| *yjjP* | G7946 | predicted inner membrane structural protein | -1.64 | 6.63 | S |
| *ynfK* | G6851 | putative dethiobiotin synthetase | -1.66 | 6.95 | H |

^a^EcoCYC accession ID. ^b^COG categories: [C] - Energy production and conversion; [H] - Coenzyme transport and metabolism; [K] – Transcription; [O] – Post-translational modification, protein turnover, chaperones; [P]- Inorganic ion transport and metabolism; [R] – General function prediction only; [S] - Function unknown and [no info] - no information was available for this gene at the time of this study.
